# Use of Vivostat PRF® in Acanthamoeba keratitis

**Published:** 2020

**Authors:** Emma Marín-Payá, Miriam Rahhal-Ortuño, Marina Aguilar-González, Alex Samir Fernández-Santodomingo, Salvador García-Delpech

**Affiliations:** * Department of Ophthalmology, Hospital Universitari i Politecnic La Fe, Valencia, Spain

**Keywords:** Acanthamoeba, keratitis, Vivostat PRF®

## Abstract

We present a case of a 27-year-old contact lens male user who was diagnosed with Acanthamoeba keratitis. Given the inefficiency of medical treatment and high risk of corneal perforation, we decided to use Vivostat PRF®, with satisfactory results. To our knowledge, this is the first described case in medical literature in which Vivostat PRF® is used as part of Acanthamoeba keratitis treatment.

## Introduction

Acanthamoeba is a protozoon that is ubiquitous in air, dust particles, water and upper respiratory tract. Its life cycle is based on a cystic state, which is very resistant and can turn into a trophozoite state when correct ambient conditions are given, which is capable of breaking through and destroying tissues [**1**,**2**]. 

Symptoms of patients with Acanthamoeba keratitis include blurry vision and pain, which may be not proportional to the severity of clinical signs. Initial alterations observed are superficial punctate keratopathy, epithelial pseudodendrites and perineural infiltrates. On a second stage, patients develop a corneal ulcer with an annular infiltrate and a marked inflammatory reaction in anterior chamber with hypopyon. Limbitis is also characteristic in affected patients [**1**,**2**]. 

Acanthamoeba keratitis is more frequent in contact lens users [**2**].

It is especially important to assure a correct diagnosis of Acanthamoeba keratitis in an early phase given that the prognosis is more favorable if early diagnosis and treatment are established. In fact, it is convenient to begin treatment with diamides and biguanides with the slightest doubt, even before laboratory’s diagnosis confirmation [**1**,**2**]. 

## Case report

A 27-year-old soft contact lens Caucasian male user with no medical history of interest, referred intense pain, photophobia, tearing and ocular redness in his left eye over the past four weeks. He had been previously treated with topical erythromycin, ciprofloxacin, tobramycin and dexamethasone with no symptomatic improvement. He admitted to have given contact lens an inappropriate use, as he wore them for over 12 hours each day, he showered with them on and even swam in rivers and swimming pools while wearing them. 

**Fig. 1:**
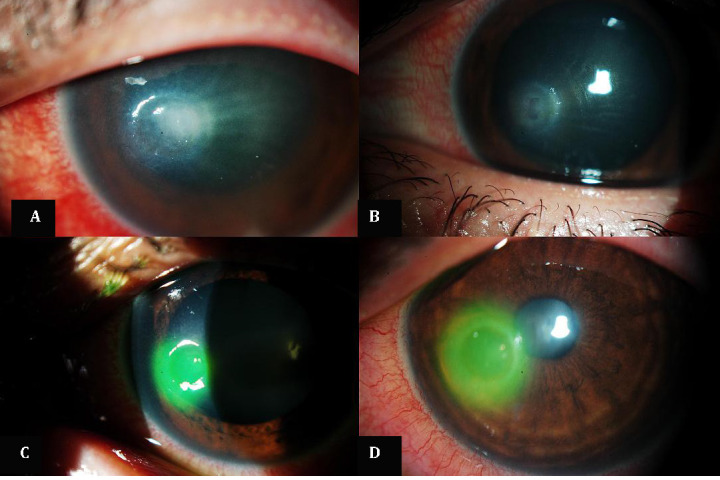
**A,B** Conjunctival hyperaemia and annular infiltration with radial perineuritis; **C,D** Eight weeks after diagnosis; improvement in corneal transparency and no radial perineuritis

His visual acuity was 1.0 and 0.1 in his right and left eye respectively according to Snellen’s scale. Intraocular pressure and pupillary light reaction were normal in both eyes. Anterior segment biomicroscopy of his left eye showed conjunctival hyperaemia, diffuse corneal decompensation and a paracentral annular infiltration with radial perineuritis (**Fig. 1A,B**). Fundus examination revealed no significant findings. Right eye examination showed no alterations. 

Acanthamoeba keratitis was suspected, corneal scraping was carried out and PCR, Gram analysis and sample cultures were requested. Empirical treatment with topical 0.1% Brolene®, 0.02% chlorhexidine, vancomycin, ceftazidime and atropine were started. A favorable initial response was observed as ten days after starting this treatment visual acuity in his left eye had improved to 0.6. Topical fluorometholone was then added. 

PCR results were positive for Acanthamoeba, Klebsiella, Serratia and S. Aureus.

Treatment was not modified due to the patient’s positive response.

The follow-up was at eight weeks after he showed a significant clinical improvement. Conjunctival hyperaemia, stromal haze and anterior chamber reaction had noticeably decreased and visual acuity was 0.8 (**Fig. 1C,D**). Treatment was therefore slowly tapered down over the following weeks. Weekly follow-up check-ups were carried out.

**Fig. 2:**
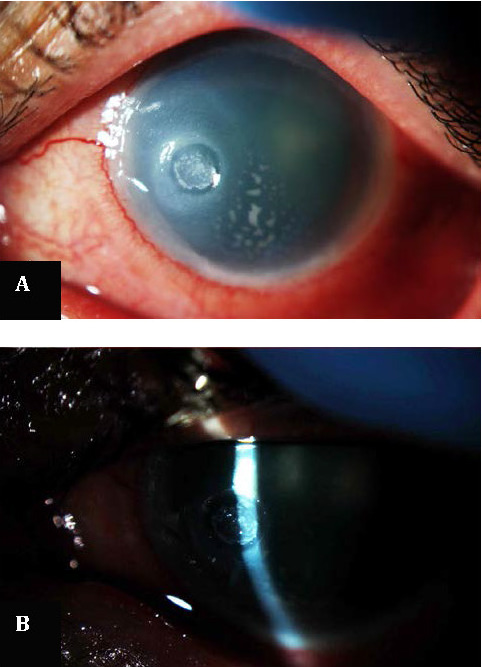
Five months into treatment; loss of corneal transparency, ulcer thinning and endothelial precipitates

Another follow-up review took place five months after he showed a drastic clinical deterioration. Anterior segment biomicroscopy revealed diffuse corneal transparency loss with endothelial deposits affecting inferior hemicornea, ulcer thinning, intense anterior chamber reaction and cataract formation (**Fig. 2**). Corneal scraping was repeated to rule out overinfection and autologous serum, topical linezolid and oral valaciclovir were empirically added to the initial treatment. Cultures then showed Pseudomona aeruginosa growth. 

**Fig. 3:**
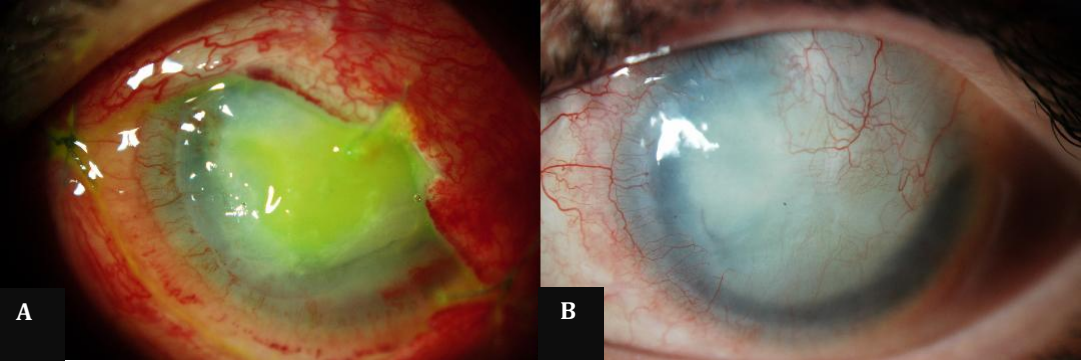
**A** Vivostat PRF® over the cornea with absorbable sutures; **B** Six weeks after Vivostat PRF®; noticeable inflammatory activity decrease, corneal leukoma and neovascularization and white cataract

Over the following two weeks, corneal thinning increased, therefore underlying the existence of a significant perforation risk. Having no corneal donor, the amniotic membrane was used to cover the corneal defect, with unsatisfactory results. Two weeks later we decided to add Vivostat PRF® in order to increase the corneal resistance to perforation and to favor its regeneration (**Fig. 3A**).

6 weeks into Vivostat PRF® treatment, corneal ulceration was healed leaving behind a dense residual leukoma and inflammatory activity ceased (**Fig. 3B**). Our patient is currently waiting for combined keratoplasty and cataract surgery.

## Discussion

Although there is no protocolized treatment for Acanthamoeba keratitis, in most cases topical treatment with 0.1% diamidines, 0.02% biguanides and low dose corticoids is initially used [**3**]. 

Vivostat PRF® is a fibrin sealant platelet-rich membrane, which aids tissue regeneration and is obtained from the patient’s own blood after carrying out high-speed centrifugation. The membrane is then sutured over the area-to-treat and fibrin polymerization immediately takes place over the defect covered by the membrane [**4**-**6**]. In our case, we sutured the fibrin compound to the cornea using absorbable sutures (**Fig. 3**).

Vivostat PRF® has been used in different medical specialties such as traumatology, cardiology, maxillofacial surgery, digestive tract surgery, urology, plastic surgery, otorhinolaryngology and neurology during surgical interventions in order to aid tissue regeneration [**6**-**9**]. To our knowledge, this is the first described case in medical literature in which Vivostat PRF® is used in Acanthamoeba keratitis treatment.

In our case, adding Vivostat PRF® to the classic treatment of Acanthamoeba keratitis was the key to increase corneal resistance and ease its regeneration process. 

**Conflict of interest**

Authors declare no conflict of interest.

**Sources of funding**

There are no funders to report for this submission.

**Ethical Clearance for study**

A written consent was gathered from the patient in order to obtain and publish these images.

**Ethical Statement**

Our study did not require an ethical board approval because it did not contain human or animal trials.
